# Determination of systemic inflammatory biomarkers in multiple sclerosis

**DOI:** 10.5937/jomb0-45083

**Published:** 2024-04-23

**Authors:** Maša Sladojević, Stanislava Nikolić, Željko Živanović, Svetlana Simić, Lorand Sakalaš, Igor Spasić, Branislava Ilinčić, Velibor Čabarkapa

**Affiliations:** 1 University of Novi Sad, Faculty of Medicine, Novi Sad; 2 Clinical Center of Vojvodina, Novi Sad; 3 Department of Laboratory Diagnostics Medlab, Novi Sad

**Keywords:** multiple sclerosis, neutrophil-to-lymphocyte ratio, C reactive protein, neurofilaments, growth differentiation factor 15, multipla skleroza, odnos neutrofila i limfocita, C reaktivni protein, neurofilamenti, faktor diferencijacije rasta 15

## Abstract

**Background:**

Multiple sclerosis (MS) is one of the most common demyelinating diseases of the central nervous system. We aimed to investigate serum and cerebrospinal fluid levels of different laboratory inflammatory biomarkers in patients with MS.

**Methods:**

A total of 120 subjects participated in the study, 60 of whom were diagnosed with MS, 30 with the final diagnosis of non-inflammatory diseases of the central nervous system (CNS), and 30 healthy subjects representing the control group. Regarding the progression of radiological findings after 2 years from the initial diagnosis, the MS group was divided into stationary radiological findings (n=30) and radiologically proven disease progression (n=30). In all patients, we analyzed levels of laboratory inflammatory biomarkers: C reactive protein (CRP), Neutrophil-to-lymphocyte ratio (NLR), Growth differentiation factor 15 (GDF15) in serum samples, and neurofilaments (NFs) in cerebrospinal fluid (CSF). NFs and GDF15 were analyzed initially, while CRP and NLR values were analyzed initially and after two years.

**Results:**

We found statistically lower GDF15 values and initial CRP values in the MS group regarding the group with non-inflammatory diseases of the CNS (p<0.0001). On the other side, we determined a significant elevation of laboratory markers CRP and NLR, initially and after a two-year period, in the MS subgroup with the progression of magnetic resonance imaging (MRI) findings (p<0.0001 and p=0.050, respectively). Also, we found a positive correlation between CRP and NFs (r=0.243, p=0.04), as well as a positive correlation between CRP and GDF15 in patients with MS (r=0.769, p<0.0001).

**Conclusions:**

We found a significant elevation of laboratory markers of systemic inflammation, CRP, and NLR in MS patients who developed disease progression based on MRI findings. There is a need for further studies to validate current parameters to be considered as useful markers of MS activity and disability.

## Abstract (sr)

**Uvod:**

Multipla skleroza je jedna od najčešćih demijelinizujućih bolesti centralnog nervnog sistema. Cilj rada je bio da se ispitaju vrednosti različitih laboratorijskih inflamatornih biomarkera u serumu i cerebrospinalnoj tečnosti kod pacijenata sa multiplom sklerozom (MS).

**Metode:**

U istraživanju je učestvovalo ukupno 120 ispitanika, od kojih je 60 sa dijagnozom MS, 30 sa konačnom dijagnozom neinflamatornih bolesti centralnog nervnog sistema (CNS) i 30 zdravih ispitanika koji su činili kontrolnu grupu. U pogledu progresije radioloških nalaza nakon 2 godine od inicijalne dijagnoze, MS grupa je podeljena u podgrupe: sa stacionarnim radiološkim nalazom (n=30) i sa radiološki dokazanom progresijom bolesti (n=30). Kod svih pacijenata smo analizirali vrednosti laboratorijskih inflamatornih biomarkera: C reaktivni protein (CRP), NLR (odnos neutrofila i limfocita), faktor diferencijacije rasta 15 (GDF15) u uzorcima seruma i neurofilamenti (NFs) iz cerebrospinalne tečnosti (CSF). Vrednosti NFs i GDF15 su analizirane inicijalno, dok su vrednosti CRP i NLR analizirane inicijalno i nakon dve godine.

**Rezultati:**

Rezultati studije ukazuju na statistički niže vrednosti GDF15 i vrednosti CRP inicijalno merenog u grupi sa MS u odnosu na grupu sa neinflamatornim oboljenjima CNS (p<0.0001). Sa druge strane, utvrdili smo signifikantno više vrednosti CRP i NLR, inicijalno i nakon dvogodišnjeg perioda, u MS podgrupi sa progresijom MRI nalaza (p<0.0001 i p=0,050, respektivno). Takođe smo utvrdili pozitivnu korelaciju CRP i NFs (r=0,243, p=0,04), kao i pozitivnu korelaciju CRP i GDF15 kod pacijenata sa MS (r=0,769, p<0.0001).

**Zaključak:**

Kod pacijenata sa dijagnozom MS kod kojih je došlo do progresije bolesti na osnovu nalaza MRI uoćava se značajno povećanje laboratorijskih markera sistemske inflamacije, CRP i NLR. Postoji potreba za daljim ispitivanjima kako bi se navedeni parametri potvrdili korisnim markerima aktivnosti i progresije obolelih od multiple skleroze.

## Introduction

Multiple sclerosis (MS) is one of the most common demyelinating diseases of the central nervous system (CNS). MS is a chronic, neuroinflammatory, neurodegenerative disease characterized by focal demyelinated lesions of the spinal cord and brain [1]. About 2.3 million people suffer from MS in the world – the highest incidence of this disease is present in North America (140 new cases per 100,000 in habitants) and in Europe (108 new cases per 100,000 inhabitants) [2] [3], while in Serbia the incidence rate is estimated to 65 new cases per 100.000 inhabitants [4].

Multiple sclerosis is most often diagnosed in relatively young people, principally in women – the incidence is highest in the age group of 25–35 years [5]. Depending on the severity of the clinical findings and the progression of the disease, nerve degeneration in MS can lead to a significant degree of disability [6] and represents a significant health problem in the most developed countries of the world [7].

The disease has a complex and still insufficiently known etiopathogenesis. It is known that the onset of MS is conditioned by an autoimmune reaction directed against the cells of the myelin sheath of the axon of neurons. However, genetic factors and infections caused by certain microorganisms also significantly initiate the disease [8] [9].

In this regard, numerous studies [10] [11] have devoted attention to identifying new and improvingexisting diagnostic tools and parameters that could more accurately indicate the severity and prognosis of the disease. Significant progress has been made in MRI technology, which has become the backbone of MS diagnostics and today enables monitoring of the disease in its earliest stages [10]. Cerebrospinal fluid analysis still represents the gold standard in presenting immunological and biochemical events in the CNS during the disease. For this reason, many studies aimed to enable the data obtained from the cerebrospinal fluid analysis to provide the best possible insight into the etiopathogenesis of MS and expand the range of relevant parameters for diagnosing this disease.

Today, it is known that MS is an autoimmune disease [12] that occurs in genetically predisposedindividuals, although the pathogenic pathways leading to MS are not well understood. In general, the pathophysiological mechanisms of MS are multifactorial; first of all, genetic and environmental factors play an essential role in disease pathogenesis [8].

CNS inflammation is the main factor of MS disease pathogenesis [9]. Cellular elements of innateand adaptive immunity (macrophages, T and B lymphocytes) are responsible for the inflammatoryprocesses. One of the leading causes of inflammation is the interaction between those cells, mainly by producing many different inflammatory and anti-inflammatory cytokines, which have a potential role in myelin sheath and axon damage processes [1]. Self-reactive T cells specific for myelin proteins are the major mediators in this process, initiating an inflammatory cascade that leads to demyelination [13]. In patients with MS, activated T-cells in the peripheral blood, together with the majority of adhesive molecules, lead to blood-brain barrier damage, and they can trigger a central immune response, which leads to the initiation of demyelination [14].

Neutrophils have a key role in the innate immune response through the secretion of proinflammatory mediators, releasing of lytic enzymes, activation of antigen-presenting and T cells, as well as pathogen clearance by phagocytosis [15] [16]. Therefore, the increase in neutrophile count can be related to the occurrence and severity of inflammation. The function of neutrophils that can contribute to the development of CNS autoimmunity is still unknown. The neutrophil-to-lymphocyte ratio (NLR) in peripheral blood has been proposed as a potential systemic inflammatory marker in several diseases, given that NLR represents the balance between neutrophil and lymphocyte levels [17] [18].

Growth differentiation factor 15 (GDF15) is a member of the transforming growth factor super-family, and it is secreted by cells as a response to varpious stressors. The receptors for GDF15 areexpressed in the brain, where its activation results in a wide range of responses. Chronic aberrant immune responses against self-antigens are characteristic of autoimmune diseases that result in immune cells turning against host tissues. In certain autoimmune diseases, high values of GDF15 are observed, which favors the induction of GDF15 in inflammatory conditions. Still, MS GDF15 was only elevated in a small subset of patients characterized by a stable course of disease with no relapse [19].

Neurofilaments (NFs) are the main components of the axonal cytoskeleton and today are considered responsible for nerve structural support and velocity conduction in myelinated fibers [20]. Given that during axon damage NFs are released into the extracellular fluid, more attention is focused on measuring the concentration of NFs in body fluids, primarily in CSF, where it can be a valuable biomarker of neuroinflammation. Neurofilaments consist of heavy (NF-H), medium (NF-M), and light (NF-L) chains, and their detection in CSF and blood samples has been the subject of interest in several neurological diseases [21]. In patients with MS, NFs were examined for the established diagnosis and biomarker of disease activity and response to medication.

Concerning the above and because the etiopathogenesis of multiple sclerosis is based on low-grade inflammation, there is a need to determine individual parameters of inflammation on the one hand and their joint influence on the course and prognosis of the disease. Also, bearing in mind that the laboratory diagnosis is insufficient in terms of the diagnosis of this disease, it is necessary to make an effort to obtain crucial information based on the existing laboratory parameters.

### Aim

In this study, we aimed to investigate levels of different laboratory inflammatory biomarkers such as CRP, NLR, and GDF15 in serum samples and levels of NFs in CSF in patients with MS.

## Materials and methods

This retrospective study was performed at the University Clinical Center of Vojvodina after access to the medical documents and approvals of the Ethics Committee of this institution.

The retrospective study included a total of 120 patients hospitalized in the period from 2019 to 2021 at the Clinic for Neurology of the University Clinical Center of Vojvodina. The patients were divided into three groups. The first group consisted of patients diagnosed with MS (n=60), the second group consisted of patients suffering from non-inflammatory diseases of the CNS (n=30), and the control group consisted of 30 healthy subjects. MS patients were divided into two subgroups regarding the progression of radiological findings 2 years from the initial diagnosis of MS, into a group of patients (n=30) with stationary findings and a group of patients (n=30) with radiologically proven disease progression. A progression was defined as the occurrence of a new demyelinating lesion detected by using MRI. The MS diagnosis was confirmed according to the revised 2017 McDonald criteria [22]. All patients were admitted to the hospital with the first symptoms, or the first attack of MS. Disease severity was determined at the time of blood collection by using the Expanded Disability Status Scale (EDSS). All MS patients were EDSS≤5. Regarding the medications, all MS patients were on interferon therapy protocol.

To avoid confounding factors, patients with acute infections, active or chronic inflammatory diseases, other autoimmune diseases, other neurological diseases, and a history of malignancy are excluded from the study.

### MRI

In performing brain MRI, a standard protocol was considered and followed [23]. Lesions were defined as active if contrast enhancement was noted on a T1-weighted sequence following an injection of a single dose of gadolinium-based contrast material.

### Laboratory parameters

Blood samples were collected after 12 hours of fasting, and blood samples were collected from the participants and stored at -80°C for further analysis. The venipuncture procedure was carried out according to standardized protocol and references [24].

The lumbar puncture procedure was performed in accordance with the description in the relevant literature, adhering to antisepsis measures [25].

Hematological parameters (neutrophils, lymphocytes, and NLR) were evaluated by drawing venous blood samples into tubes containing dipotassium EDTA as an anticoagulant. Complete blood count was analyzed on the hematology counter Sysmex XN100 using commercial reagents, according to the manufacturer’s recommendations (Sysmex, XN1000, Germany, Europe). Commercial control materials were used for quality control protocol.

The value of NLR was calculated as the ratio between neutrophils and lymphocytes [26] [27]. The level of CRP was determined by standard biochemical method on a biochemical analyzer Abbott Architect c 8000 (Abbott Park, Illinois, USA) by immunoturbidimetric method. The reference range for CRP in adults is <10 mg/L. Commercial control materials were used for quality control protocol.

In MS patients, CRP and NLR were determined at the admission to the hospital as well as after 2 years of diagnosis.

The level of NFs in cerebrospinal fluid was determined by the ELISA method using the Neurophilament (pNf-H) ELISA test (Euroimmun, L beck, Germany) at automated Euroimmun Analyzer I-2P. According to the manufacturer’s recommendation, control materials were used (Control 1 and Control 2). Intra and inter-assay coefficients of variability (CV%) were <5 and <12, respectively. The measuring range was 0.6–11.5 ng/mL, with a detection limit of 0.027 ng/mL. Expected values for CSF samples are negative ≤ 0.69 ng/mL, borderline <0.69 to <1.52 ng/mL, and positive 1.52 ng/mL. Hemo lytic, lipaemic, and icteric serum samples showed no influence on the result up to a concentration of 10 mg/mL hemoglobin, 20 mg/mL triglycerides, and 0.4 mg/mL bilirubin in this ELISA assay.

The level of GDF15 in serum was determined by ELISA method using the Quantikine Elisa Human GDF-15 Immunoassay, research use only (Bio-Techne, Abingdon, United Kingdom) on ELISA Reader. Intra and inter-assay coefficients of variability (CV%) were <3 and <6, respectively. The measuring range was 337–1060 pg/mL, with a 2 pg/mL detection limit. This assay is designed to eliminate interference by other factors present in biological samples. Until all factors have been tested in Quantikine Immunoassay, the possibility of interference cannot be excluded.

### Statistical analysis

Complete statistical analysis was done using the Data Analysis Excel (Microsoft Corp, Redmond, WA) statistical software and MedCalc® Statistical Software version 20.217 (MedCalc Software Ltd, Ostend). The Student’s t-test was used to compare the two independent groups according to their distribution state. To examine statistically significant differences, 2 levels of measurement were applied – multivariate and univariate analysis of variance for repeated measurements (MANOVA and ANOVA). Pearson correlation was used to determine the relationship between continuous variables. The statistical significance level was determined as 0.05. Tables and graphs display the results.

## Results

In the current study, we examined 60 patients with definite MS diagnoses. About 60% (n=36) of the cases were female, and the rest, 40% (n=24), were male, and their age ranged from 18 to 55 years with a mean age (±SD) of 38.58 (±10.38). All patients were further divided into two subgroups regarding the progression of radiological findings after 2 years from the diagnosis of MS.

All groups were age and gender-matched.

As well as in the healthy control group, CRP values in the MS group were within the reference range. We found statistically lower CRP values in the MS group regarding the group with non-inflammatory diseases of the CNS (*p*<0.0001; Table 1).

**Table 1:** The level of the investigated biomarkers in serum and CSF. NA: not applicable; sCRP – C reactive protein in serum; sNLR – Neutrophil-to-lymphocyte ratio in serum; cNFs – Neurofilaments in cerebrospinal fluid; GDF15- Growth differentiation factor 15 in serum; !significant MS group regard to healthy control; *significant MS group regard to group with non-inflammatory diseases; ? significant healthy control group with regard to the group with non-inflammatory diseases

	MS group<br>(n=60)	Group with non-inflammatory<br>diseases of the CNS (n=30)	Healthy control group<br>(n=30)	*p*-value
sCRP	0.80 (0.52–1.00)	6.90 (1.31–18.53)	1.60 (1.00–2.50)	*0.000
sNLR	2.12 (1.76–2.66)	2.23 (1.57–3.62)	1.24 (0.97–1.46)	!?0.000
cNFs	0.13 (0.02–0.18)	0.15 (0.11–0.21)	NA	0.830
sGDF15	143 (122–178)!	300 (210–368)	NA	0.000

However, NLR values were significantly higher in both MS and the non-inflammatory disease group than in the healthy control group (*p*<0.0001; Table 1).

The levels of NFs in CSF were lower in the MS group regarding the group with non-inflammatory diseases of the CNS but without statistical significance (*p*=0.83; Table 1).

Results of our study indicate that the values of GDF15 were statistically lower in the MS group regarding the group with non-inflammatory diseases of the CNS (*p*<0.0001; Table 1).

As we divided the MS group into whether they have or have not developed MRI signs of progression of the disease during a period of two years, we examined CRP and NLR values. According to the results of this study, initial as well as CRP values after two years were statistically lower in the MS subgroup with stationary MRI findings in regards to the MS subgroup with new radiological findings (*p*=0.042 and *p*<0.0001, respectively; Table 2). Similar results were observed for NLR values (*p*=0.021 and *p*<0.0001, respectively; Table 2).

**Table 2:** The level of serum CRP and serum NLR in the MS group. sCRP – C reactive protein in serum; sNLR – Neutrophil-to-lymphocyte ratio in serum

	MS subgroup with stationary<br>MRI findings (n=30)	MS subgroup with the progression<br>of MRI findings (n=30)	p-value
sCRP	0.10 (0.10–0.10)	0.95 (0.57–1.95)	0.042
sCRP after 2 years	0.10 (0.10–0.10)	4.25 (3.00–6.00)	0.000
sNLR	1.71 (1.34–2.53)	2.93 (2.02–3.15)	0.021
sNLR after 2 years	1.77 (1.62–1.94)	3.86 (2.74–4.83)	0.000

During two years, the CRP and NLR values did not significantly differ in the first MS subgroup (*p*<0.0001 and *p*=0.905, respectively). On the other side, we determined a significant elevation of both laboratory markers, CRP and NLR, in the MS sub-group with the progression of MRI findings (*p*<0.0001 and *p*=0.050, respectively).

Results of our study indicate that values of neurofilaments are higher in the group of MS patients with new radiological findings in regards to the group with stationary MRI findings but without statistical significance (*p*=0.820; Table 3). Also, the GDF15 values were higher in the MS group of patients with stationary MRI findings than those with new radiological findings but without statistical significance (*p*=0.782; Table 3).

**Table 3:** The level of investigated biomarkers in serum and CSF in the MS group. NFs – neurofilaments in cerebrospinal fluid; GDF-15 – Growth differentiation factor 15 in serum

	MS group with stationary<br>MRI findings (n=30)	MS group with progression<br>of MRI findings (n=30)	p-value
cNFs	0.20 (0.11–1.29)	0.30 (0.27–1.42)	0.820
sGDF15	183 (25–760)	172 (46–460)	0.782

The results of our study indicate the presence of a positive correlation between CRP and neurofilaments, as well as a positive correlation between CRP and GDF15 in patients with MS (Table 4).

**Table 4:** Linear correlation analysis between baseline CRP, neurofilaments, and GDF15 in patients with MS. CRP – C reactive protein; NFs – neurofilaments; GDF15 – Growth differentiation factor 15

n=60	CRP	
NFs	R	p
	0.760	0.000
GDF15	0.243	0.040

## Discussion

Systemic inflammation can be the leading cause of neurodegeneration and can play an essential role in the pathogenesis of MS. The crucial pathophysiological mechanism for disease onset is producing proinflammatory cytokines and activating innate and adaptive immune cells, leading to an inflammatory response within the CNS [26].

Our study’s results indicate that CRP values in the MS group are within the reference range. Still, the NLR values on the first time of measuring are statistically higher in the MS group regarding healthy control. Similar results were found in other studies [26] [27]. Hasselbalch’s and Demirci’s studies found increased systemic inflammation parameters, as measured by NLR in MS patients compared to controls. Elevated NLR can indicate hematopoietic disproportions toward increased myeloid innate immune system cell production regarding the dysregulated adaptive immune system.

For a more detailed analysis of the most frequently applied laboratory parameters of systemic inflammation (CRP and NLR), we divided the group of patients with multiple sclerosis into two subgroups, depending on clear signs of disease progression, determined by MRI. CRP and NLR values were statistically lower in the first subgroup of patients and did not change significantly after two years. However, the values of these two parameters significantly increased in the subgroup of patients with multiple sclerosis in whom MRI signs of progression were detected. Similar to our findings, higher NLR during relapse of the disease was reported in the Naegele et al. [28] study, which can be explained by high values of neutrophils in the inflammation associated with the relapse of the disease.

Our study’s results indicate no statistical significance in the NF values in the MS group regarding the group with non-inflammatory diseases of the CNS. Certain studies indicate elevated values of neurofilaments in various non-inflammatory neurological diseases [29]. Results from another study suggested that NF levels could be elevated during all stages of MS, suggesting that NFs are associated with various pathological processes involved in MS, reflecting disease activity and progression [30]. According to our results, NF values were slightly higher in the MS group with new radiological findings in the group with patients with stationary MRI findings. In several studies, elevated values of NFs in patients with first attack of MS were a predictor of future relapses [31] [32]. Also, some other studies suggested that NFs have predictive value for future MRI brain atrophy [33] [34]. It is important to know that NF-L and NF-H levels do not always correlate directly. That can be explained due to differences in protein stability and sensitivity of different assays. It is thought that NF-L assay is associated with the initial inflammatory stage of MS, as it detects early acute and correlates weaker with disability progression.

On the other hand, the NF-H assay is considered a marker of neurodegeneration since it highly corresponds with the axonal damage during disease progression [35]. Our study examined levels of NF-H, and the results agree with other studies [36] [37]. We also found an increased value of NF levels in patients with new lesions detected by MRI.

Serum GDF15 values under physiological conditions are low. In states of various tissue damage, there is an increased expression of these factors. Certain studies have shown that elevated serum values of GDF15 may indicate subclinical CNS tissue damage in MS patients. Firstly, compared to the group with non-inflammatory diseases, the GDF15 levels were statistically lower in the MS patients. Also, in this study, GDF15 values were statistically higher in the MS group with stationary MRI findings versus the group with patients with new radiological findings. That result is following Amstad et al. study [19]. Increased GDF15 concentrations may reflect an endogenous anti-inflammatory mechanism in patients with stable disease. Also, we found a positive correlation between CRP and GDF15 in patients with MS, which favors the existence of the inflammatory process.

Systemic inflammation can be the leading cause of neurodegeneration and can play an important role in the pathogenesis of MS. The most important pathophysiological mechanism for the onset of disease is producing proinflammatory cytokines and activating innate and adaptive immune cells, leading to an inflammatory response within the CNS [37]. Considering the facts above, inflammatory markers can be used as markers of MS activity and disability. Numerous studies have linked different laboratory inflammatory biomarkers in serum and cerebrospinal fluid in recent years with MS disease prognosis and patient activity. Additionally, this will allow for larger studies to determine and validate cut-off values of inflammatory biomarkers and find new accurate, accessible, validated, and minimally invasive prognostic and diagnostic biomarkers to closely monitor disease activity, disability progression, and therapeutic response. Further studies with larger cohorts are needed to validate current and new biomarkers to become solid enough to be incorporated into diagnostic criteria and become widespread enough to become part of routine algorithms in diagnosing MS.

### Limitations

The present study has some limitations, many of which are related to the retrospective design and small sample size. The results build on simple data from routine laboratory tests and, therefore, may be biased toward the availability of specific laboratory data.

## Conclusion

According to the study results, elevation of laboratory markers of systemic inflammation, CRP, and NLR in MS were found in patients who developed disease progression based on MRI findings. There is a need for additional studies to indicate current parameters as valuable markers of MS activity and disease progression.

## Dodatak

### Author declaration

Authors certify that the manuscript represents a valid piece of work and neither this manuscript nor one with substantially similar content under named authorship has been published or is being considered for publication elsewhere. The authors have participated in the research and the shaping of the manuscript.

### Conflict of interest statement

All the authors declare that they have no conflict of interest in this work.
